# Supplementary material for: Comparative multiomics analysis of cell physiological state after culture in a basket bioreactor
Source: Sci Rep. 2022 Nov 23;12:20161. doi: 10.1038/s41598-022-24687-4 (PMC9686226; doi:10.1038/s41598-022-24687-4)
Supplement: Supplementary file 1 — Supplementary Information 1. [file 41598_2022_24687_MOESM1_ESM.zip › raw data/Proteomic raw data/6.KEGGenrichment/EG.vs.CG/ALL/pathway/src/05200.html]

map05200


- K06237, K05637, K06240, K06241, K05636, K06243, K06244, K06245, K05635, K06246, K06247, K05717

- Down regulated proteins

A0A0D9R486(Description:Collagen type IV alpha 3 chain;Gene:COL4A3;log2FC:-1.4852)

A0A0D9RY11(Description:Collagen type IV alpha 2 chain;Gene:COL4A2;log2FC:-1.7446)

A0A0D9RY12(Description:Collagen IV NC1 domain-containing protein;Gene:COL4A1;log2FC:-1.2021)


A0A0D9R9X4(Description:Fibronectin;Gene:FN1;log2FC:-1.1754)

- K02432, K02235, K02329, K02354, K02375, K02376, K02842

- Up regulated proteins


A0A0D9SCP4(Description:Uncharacterized protein;Gene:FZD1;log2FC:1.4940)

- K04707

- Up regulated proteins

A0A0D9R1V2(Description:E3 ubiquitin-protein ligase CBL;Gene:CBLB;log2FC:1.1276)

- K02158

- Up regulated proteins

A0A0D9R491(Description:Uncharacterized protein;Gene:BAD;log2FC:inf)

- K02158

- Up regulated proteins

A0A0D9R491(Description:Uncharacterized protein;Gene:BAD;log2FC:inf)

- K06618

- Up regulated proteins

A0A0D9RYS3(Description:Uncharacterized protein;Gene:RB1;log2FC:1.1115)

- K00799

- Up regulated proteins


A0A0D9R7G0(Description:Glutathione transferase;Gene:MGST1;log2FC:1.2430)

Close
